# Machine learning-based screening of an epithelial-mesenchymal transition-related long non-coding RNA signature reveals lower-grade glioma prognosis and the tumor microenvironment and predicts antitumor therapy response

**DOI:** 10.3389/fmolb.2022.942966

**Published:** 2022-08-26

**Authors:** Nan Wang, Xin Gao, Hang Ji, Shuai Ma, Jiasheng Wu, Jiawei Dong, Fang Wang, Hongtao Zhao, Zhihui Liu, Xiuwei Yan, Bo Li, Jianyang Du, Jiheng Zhang, Shaoshan Hu

**Affiliations:** ^1^ Department of Neurosurgery, Emergency Medicine Center, Zhejiang Provincial People’s Hospital, Affiliated to Hangzhou Medical College, Hangzhou, China; ^2^ Department of Neurosurgery, The Second Affiliated Hospital of Harbin Medical University, Harbin, China; ^3^ Department of Neurosurgery, West China Hospital, Sichuan University, Chengdu, China; ^4^ Department of Neurosurgery, Taizhou First People’s Hospital, Taizhou, China; ^5^ Department of Neurosurgery, Shandong Provincial Hospital Affiliated to Shandong First Medical University, Jinan, China

**Keywords:** epithelial-mesenchymal transition, long non-coding RNAs, lower-grade gliomas, tumor microenvironment, antitumor treatment

## Abstract

Epithelial-mesenchymal transition (EMT) confers high invasive and migratory capacity to cancer cells, which limits the effectiveness of tumor therapy. Long non-coding RNAs (lncRNAs) can regulate the dynamic process of EMT at different levels through various complex regulatory networks. We aimed to comprehensively analyze and screen EMT-related lncRNAs to characterize lower-grade glioma (LGG) tumor biology and provide new ideas for current therapeutic approaches. We retrieved 1065 LGG samples from the Cancer Genome Atlas and Chinese Glioma Genome Atlas by machine learning algorithms, identified three hub lncRNAs including CRNDE, LINC00665, and NEAT1, and established an EMT-related lncRNA signature (EMTrLS). This novel signature had strong prognostic value and potential clinical significance. EMTrLS described LGG genomic alterations and clinical features including gene mutations, tumor mutational burden, World Health Organization (WHO) grade, IDH status, and 1p/19q status. Notably, stratified analysis revealed activation of malignancy-related and metabolic pathways in the EMTrLS-high cohort. Moreover, the population with increased EMTrLS scores had increased cells with immune killing function. However, this antitumor immune function may be suppressed by increased Tregs and macrophages. Meanwhile, the relatively high expression of immune checkpoints explained the immunosuppressive state of patients with high EMTrLS scores. Importantly, we validated this result by quantifying the course of antitumor immunity. In particular, EMTrLS stratification enabled assessment of the responsiveness of LGG to chemotherapeutic drug efficacy and PD1 blockade. In conclusion, our findings complement the foundation of molecular studies of LGG, provide valuable insight into our understanding of EMT-related lncRNAs, and offer new strategies for LGG therapy.

## Introduction

Lower-grade glioma (LGG), consisting of World Health Organization (WHO) grades II and III, has a better prognosis relative to glioblastoma. However, it is worth noting that LGG still has the potential for malignant transformation and strong aggressiveness (Cancer Genome Atlas Research Network, 2015; Gittleman et al., 2020). The primary treatment modality for LGG is surgical intervention with the aim of maximizing tumor resection and obtaining sufficient tissue for detailed molecular and genetic characterization (Weller et al., 2021). The WHO guidelines for the management of glioma issued in 2021 place greater emphasis on molecular diagnosis compared to the 2016 guidelines (Louis et al., 2016; Gritsch et al., 2022). This demonstrates that molecular-based research can optimize the diagnosis and treatment of LGG. Especially in the context of the breakthrough of immune checkpoint blockade (ICB) for tumor treatment, in-depth molecular studies can not only reveal the malignant mechanism of LGG but also identify patients who are sensitive to antitumor therapy.

Epithelial-mesenchymal transition (EMT), is the biological process by which epithelial cells are transformed into cells with a mesenchymal phenotype by a specific procedure. In the context of tumor formation, EMT confers a variety of characteristics associated with high malignancy to individual cancer cells (Yang et al., 2020). Current studies have demonstrated the existence of multiple complex EMT regulatory cascades in gliomas that promote malignant proliferation and metastasis of tumor cells. For example, P75CUX1 regulates EMT *via* β-catenin (Xu et al., 2021), and miR-19a/b promotes EMT by regulating the SEPT7-AKT-NF-κB pathway (Wang et al., 2021). In addition, restriction of ferritin light chain expression modulates AKT-SK3β-β linked protein signaling, which in turn inhibits EMT and temozolomide resistance in glioma (Liu et al., 2020). Consequently, EMT is considered a key regulator of tumor metastatic progression and therapeutic resistance, including surgical resection, chemotherapy, radiotherapy, and targeted therapy (Dongre and Weinberg, 2019). Therefore, targeting EMT as a strategy for LGG will have broad and far-reaching effects.

Notably, lncRNAs, as one of the major regulators in EMT development, can be involved in the dynamic process of EMT at different levels through a variety of complex regulatory networks (Liu et al., 2021). In hepatocellular carcinoma, the amplification of lncRNA ZFAS1 promoted the expression of EMT-related genes (Li et al., 2015). STAT3-activated lncRNA HOXD-AS1 suppressed the expression of SOX4 by blocking miR-130a-3p, which eventually upregulated EMT-related signaling targets and similarly enhanced the migration and invasion of hepatocellular carcinoma (Wang et al., 2017). Since most studies to date have focused only on the specific functions of lncRNAs, our overall understanding of EMT-related lncRNAs is limited, especially concerning the impact on LGG. Therefore, a comprehensive analysis of EMT-related lncRNAs is urgently needed. Furthermore, the understanding of how EMT is regulated to affect the tumor microenvironment (TME) is steadily increasing (Dongre and Weinberg, 2019). A study has shown that EMT-related gene expression may alter the level of T-cell infiltration, which may affect the responsiveness of immunotherapy and patient survival (Wang et al., 2018). Therefore, an in-depth analysis of the TME of EMT-related lncRNAs in LGG will provide new strategies for tumor biology and tumor therapy research.

Accordingly, we determined that EMT is significantly activated and affects the prognosis of LGG. Utilizing machine learning, three hub EMT-related lncRNAs were deeply mined and used as markers to construct an EMT-related lncRNA signature (EMTrLS) to further investigate the different features of mRNA expression profiles, clinicopathological parameters, malignant pathways, tumor metabolism, gene mutations, and tumor mutation burden (TMB) in LGG. In addition, stratified analysis of the EMTrLS scores quantified LGG immune cell infiltration and antitumor immune function. Notably, in addition to its strong prognostic value, the EMTrLS score is also intensely sensitive in predicting the efficacy of chemotherapy and treatment response to ICB in LGG patients. In conclusion, our analysis of EMT-related lncRNAs quantified the characteristics of LGG and provided a viable reference for the targeted treatment of LGG.

## Materials and methods

### Gene expression dataset

The LGG transcriptome expression profiles [Fragments Per Kilobase of exon model per Million mapped reads (FPKM) values] and corresponding clinicopathological data of The Cancer Genome Atlas (TCGA) dataset were obtained from the TCGA GDC project of the UCSC Xena data portal (https://xena.ucsc.edu/). After removing samples with no survival information or a survival time of fewer than 30 days, 475 LGG samples were finally obtained. mRNA expression profiles (FPKM) of 105 normal brain tissues from GTEx used as a control with TCGA LGG were also obtained from UCSC Xena. To make the gene expression profiles comparable across platforms, we converted the two sets of FPKM values to Transcripts Per Kilobase of exon model per Million mapped reads (TPM) values using the R package “limma” (Ritchie et al., 2015). The processed values were then fused to reduce batch effects and errors introduced by the integration process. The transcriptome expression profiles of 33 cancers were obtained also from the TCGA GDC project of the UCSC Xena data portal.

In addition, somatic mutation data (MAF format) were downloaded from the TCGA database (https://portal.gdc.cancer.gov/) for 475 LGG patients. The mutation types and frequencies of genes were analyzed and visualized using the “maftools” R software package (Mayakonda et al., 2018). The somatic mutation data were also used to calculate TMB, which can predict immunotherapy response (Chalmers et al., 2017).

The CGGA1 dataset was obtained from the CGGA 325 project in the China Glioma Genome Atlas (CGGA) database (http://www.cgga.org.cn/). The CGGA2 dataset was obtained from the CGGA 693 project. Based on the same inclusion criteria, the expression profiles and corresponding clinical data of 170 and 420 LGG samples were identified in CGGA1 and CGGA2, respectively (Supplementary Table S1). In addition, 20 CGGA nonglioma samples (Dataset ID: mRNA sequencing control) were included as controls. The data was also normalized by TPM and batch effects were removed with the aid of the R package “limma”.

### Analysis flow chart

**FIGURE 1:**
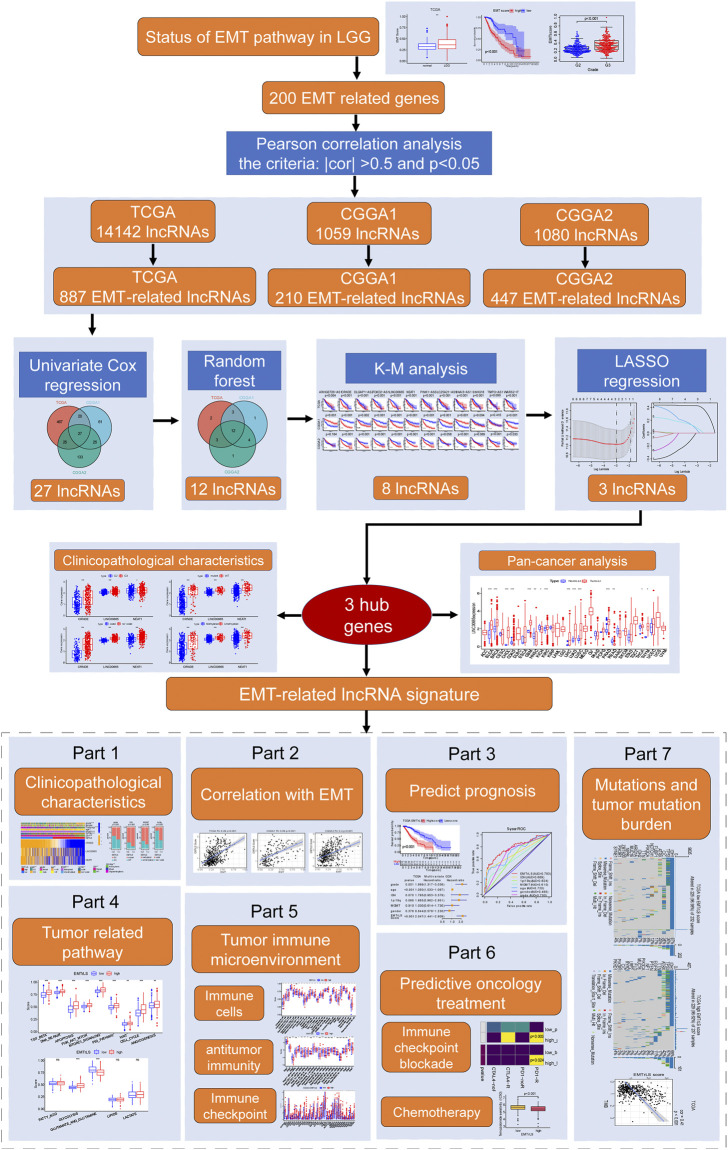
Flow chart of this study.

After data acquisition and processing, we followed the flow chart in Figure 1 for further analysis.

### Epithelial-mesenchymal transition-related LncRNAs

The TCGA, CGGA1, and CGGA2 datasets were annotated based on the Genome Reference Consortium Human Build 38 (GRCh38) annotation file from the GENCODE website (https://www.gencodegenes.org/human/). A total of 14,142, 1,059, and 1,080 lncRNAs were identified in the three datasets, respectively. We retrieved a total of 200 EMT signature genes from the Molecular Signatures Database (MSigDB, https://www.gsea-msigdb.org/gsea/msigdb/index.jsp). Based on the criteria of an absolute value of Pearson correlation coefficient >0.5 and *p*-value < 0.05, we screened 887, 210, and 447 EMT-related lncRNAs in TCGA, CGGA1, and CGGA2, respectively.

### Constructing EMTrLS

Combined with survival information, all EMT-related lncRNAs were screened by univariate Cox regression analysis, and 539, 133, and 210 lncRNAs with prognostic values were obtained in the TCGA, CGGA1, and CGGA2 datasets, respectively. Twenty-seven of these lncRNAs had significant prognostic significance in all three datasets. The random forest (RF) algorithm was used to rank the weights of the prognostic value of the 27 lncRNAs with the R package “randomForest”. Only 12 of the same gene importance values in the three datasets ranked among to the top 20, and Kaplan-Meier (K-M) survival curves showed that 8 of the 12 had a significant prognostic impact. Finally, least absolute shrinkage and selection operator (LASSO) regression analysis of the eight lncRNAs in the three datasets was performed by the R package “glmnet” to screen the biomarkers for EMTrLS. Based on the LASSO regression coefficients and expression [after log_2_ (TPM+1) transformation] of each hub gene, the following equation was used to calculate the EMTrLS score for LGG:

$$\mathrm{EMTrLS score}=\sum_{i=0}^{n}\left(\mathrm{Regression Coeffecient*Expression}\right)$$

For the stratified analysis, samples from the three datasets were grouped into EMTrLS-high and -low groups according to the median value of TCGA.

Receiver operating characteristic (ROC) curves and corresponding area under the curve (AUC) values were used to assess the predictive power of the EMTrLS score and clinicopathological characteristics on prognosis using the “survivalROC” R package. The “survival” package was used to perform univariate and multivariate Cox regression analyses to assess the independent prognostic value of EMTrLS and clinicopathological characteristics. The results were visualized with the “forestplot” package.

### Functional enrichment analysis

The R package “limma” was used to screen for differentially expressed genes (DEGs) between the EMTrLS-low and -high groups, with the implementation criteria of | log2(Fold Change) | > 1, *p* < 0.05, and FDR <0.05. Based on the R packages “clusterProfiler” and “enrichplot”, the 913 DEGs highly expressed in the high EMTrLS score group [log2(Fold Change) > 1] underwent for gene ontology (GO) [cellular component (CC), molecular function (MF), biological process (BP)] and Kyoto Encyclopaedia of Genes and Genomes (KEGG) analyses.

### Single-sample gene set enrichment analysis

The single-sample gene set enrichment analysis (ssGSEA) algorithm is calculated by rank normalization of the sample gene expression values based on genes in a known pathway and then utilization of an empirical cumulative distribution function to determine the enrichment status in that pathway (Barbie et al., 2009). With this calculation, we explored the activation status of nine classical tumor pathways and five metabolism-related pathways. The marker genes for the pathways of interest were obtained from MSigDB. ssGSEA scores were normalized to a unit distribution in the analysis, where 0 is the minimum activation value for each pathway and 1 represents the maximum.

### Immunoinformatics analysis

The “estimate” R package was used to assess the overall level of immune infiltration for each sample, ultimately providing three scores: immune score, stromal score, and estimated score (Yoshihara et al., 2013). We used two methods to assess the abundance of each type of immune cell infiltration within the tumor tissue, namely, TISIDB (http://cis.hku.hk/TISIDB) and ImmuCellAI (http://bioinfo.life.hust.edu.cn/ImmuCellAI). TISIDB is a user-friendly website containing signature genes for 28 tumor-infiltrating immune cells (Ru et al., 2019). With these signature genes, we quantified the immune infiltration of LGG samples using the ssGSEA algorithm. The ImmuneCellAI website enables online analysis and estimation of immune infiltration for each sample, including the total infiltration fraction and the level of infiltration of 24 immune cell types (Miao et al., 2020).

Tumor Immunophenotyping (TIP) (http://biocc.hrbmu.edu.cn/TIP/) is a tool that allows easy and rapid analysis of the anticancer immune process (Xu et al., 2018). It divides this process into 7 steps: step 1 tumor cell antigen release, step 2 cancer antigen presentation, step 3 stimulation and activation, step 4 immune cell transfer to the tumor, step 5 immune cell infiltration, step 6 T-cell recognition of cancer cells and step 7 killing of cancer cells. By online analysis, we obtained quantitative results of the anticancer immune process for each LGG sample.

### Predicting the responsiveness of anticancer treatments

The submap algorithm in GenePattern (https://cloud.genepattern.org/gp/pages/index.jsf) was used to predict the response of EMTrLS-high and -low cohorts to ICB treatment. The advantage of this algorithm is that it uses an unsupervised bidirectional subset projection approach that reveals similar subtypes between independent datasets (Hoshida et al., 2007). Gene expression files and information files for the immunotherapy samples used as controls were obtained from a report on melanoma (Roh et al., 2017). After obtaining the normal *p*-values and Bonferroni-corrected *p*-values, they were visualized using the R package “pheatmap”.

The R package “PRRophetic” contains the drug effects of various cell lines included in the Cancer Genome Project (CGP) database. Based on this, a ridge regression analysis was constructed to predict the IC50 value of the drug by combining LGG expression profiles and high and low EMTrLS cohort grouping information; the smaller the IC50 value was, the stronger the drug’s ability to inhibit tumor cell growth.

### Statistical analysis

Statistical analyses were performed based on using R software version 4.1.2. K-M analysis and log-rank statistical tests were used to detect differences in overall survival (OS) between the groups. The Wilcoxon test was used to compare differences in EMT, EMTrLS scores, ssGSEA scores, and gene expression. The Kruskal test was used to compare differences between histopathological subtypes. Fisher’s exact test was used to assess differences in O (6)-methylguanine DNA methyltransferase (MGMT) promoter methylation status, IDH status, WHO grade, and 1p/19q status between the low and high EMTrLS score subgroups in response to ICB treatment. *p* < 0.05 was considered to be statistically significant.

## Results

### Characteristics of Epithelial-mesenchymal transition

**FIGURE 2:**
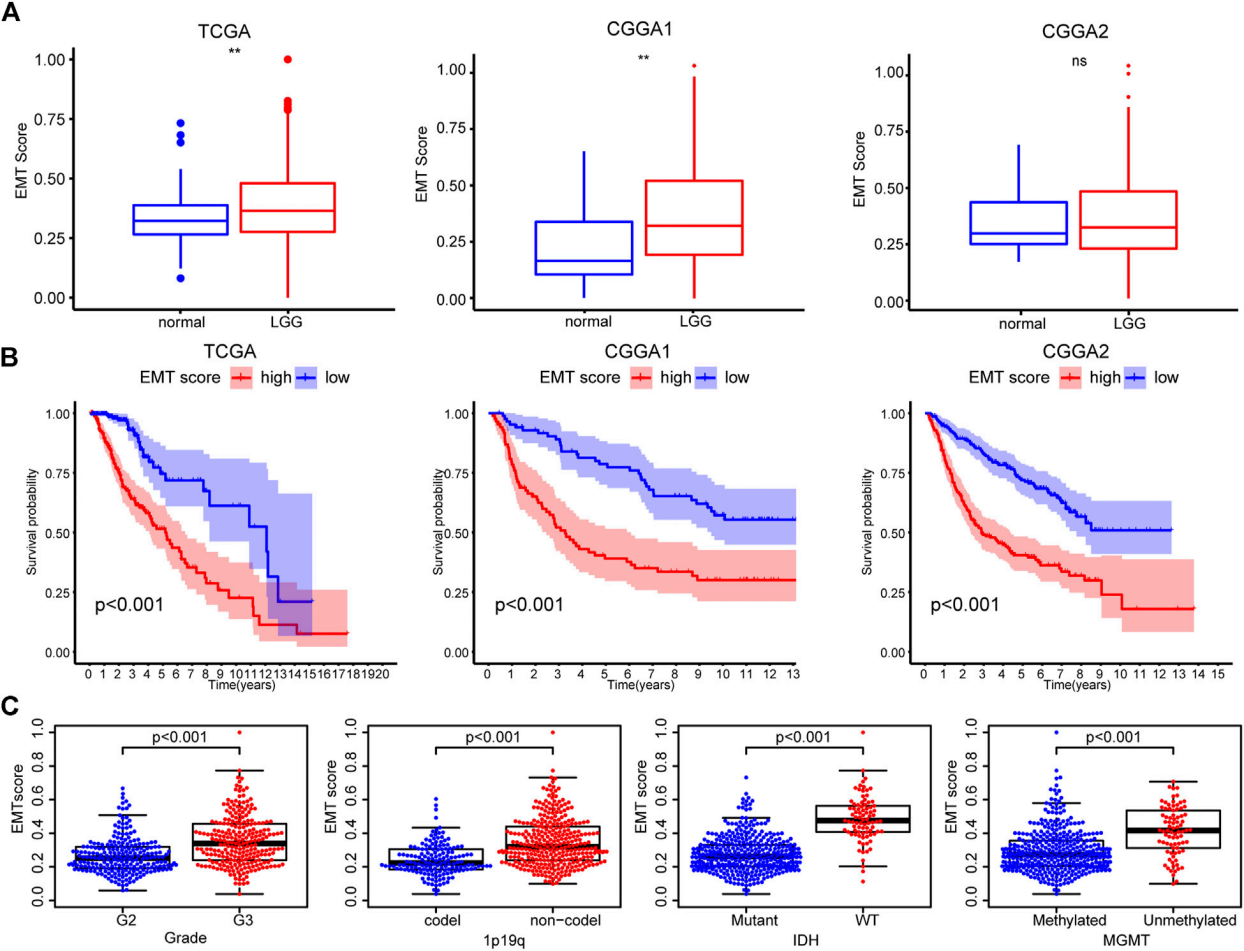
**(A)** Differences in EMT scores between nontumor tissues and LGG based on the TCGA, CGGA1, and CGGA2 datasets **(B)** K-M curves of the high and low EMT score groups **(C)** EMT scores of TCGA samples with different clinicopathological characteristics (WHO grade, 1p/19q, IDH, and MGMT status).

First, we evaluated the activation of the EMT pathway in LGG using the ssGSEA algorithm. A higher EMT score was found in LGG tissue than in normal tissue, which was validated in the CGGA1 dataset (Figure 2A). Interestingly, LGG patients with increased EMT scores had decreased OS compared with patients with low scores (Figure 2B). Moreover, samples characterized by WHO grade II, IDH mutation, and 1p19q codeletion had a significantly decreased EMT score, corroborating the association between the EMT score and poor prognosis (Figure 2C, Supplementary Figures S1A,B). The above results suggested an aberrant EMT in LGG tumor cells and an association between increased activation of EMT and enhanced malignancy of LGG.

### Machine learning screening of Epithelial-mesenchymal transition-related LncRNAs

**FIGURE 3:**
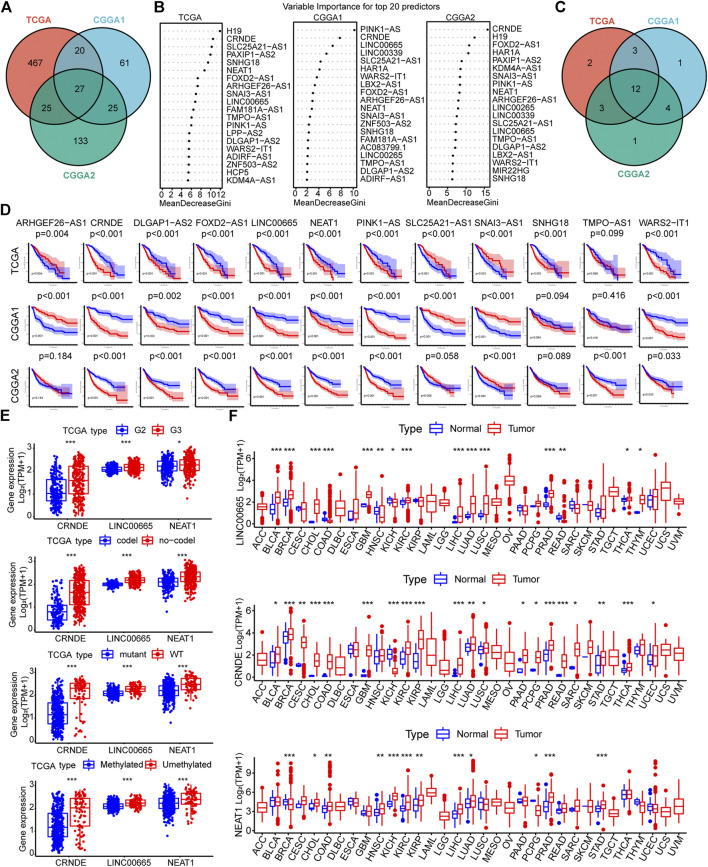
**(A)** Venn diagram showing the results of univariate Cox regression analysis of EMT-related lncRNAs in the three datasets **(B)** Importance ranking of EMT-related lncRNAs based on Gini coefficient of the random forest algorithm (top 20) **(C)** Venn diagrams of the top 20 lncRNAs in the three datasets in terms of prognostic impact **(D)** K-M curves of 12 lncRNAs. The samples were divided into two groups based on the median value of each gene expression. Red represents the high expression group, and blue represents the low expression group **(E)** Expression of the three hub lncRNAs in TCGA samples with different clinicopathological parameters (WHO grade, 1p/19q, IDH, and MGMT status) **(F)** Pan-cancer analysis of the three hub EMT-related lncRNAs (*p < 0.05, **p < 0.01, and ***p < 0.001).

Then, lncRNAs associated with the 200 EMT signature genes were identified using Pearson correlation analysis. As a result, we obtained 887, 210, and 447 EMT-related lncRNAs (| cor | > 0.5, *p* < 0.05) in the TCGA, CGGA1, and CGGA2 datasets, respectively (Supplementary Tables S2–S4). Furthermore, 539, 133, and 210 lncRNAs of prognostic significance were identified, and 27 of them were common among the three datasets (Figure 3A, Supplementary Table S5). The RF algorithm allows for feature importance assessment, and we focused on assessing how much each lncRNA contributes to the prognosis and comparing the importance of different EMT-related lncRNAs after determining the Gini mean. Notably, only 12 lncRNAs ranked in the top 20 in the three datasets based on the mean Gini decrease (Figures 3B,C). The K-M curves of 12 lncRNAs showed that the high and low expression group of CRNDE, DLGAP1-AS2, FOXD2-AS1, LINC00665, NEAT1, PINK1-AS, SNAI3-AS1, and WARS2-IT1 showed significant differences in survival time (Figure 3D). Accordingly, we performed LASSO regression to screen for significantly characterized genes again and finally identified three hub lncRNAs, namely CRNDE, LINC00665, and NEAT1 (Supplementary Figure S1C).

Furthermore, we explored the association between the expression of the three hub EMT-related lncRNAs and different clinicopathological features. The expression of the hub lncRNAs was down-regulated in samples with IDH mutation and 1p19q codeletion. CRNDE showed low expression in WHO grade II samples, while the expression of LINC00665 and NEAT1 remained stable across glioma grades in the CGGA cohorts (Figure 3E, Supplementary Figures S1D,E). In addition, the expression of the hub lncRNAs did not seem to be strongly correlated with MGMT promoter methylation status. Next, pan-cancer analysis was employed to investigate the effect of the hub lncRNAs on tumorigenesis development. The results showed that LINC00665, NEAT1, and CRNDE were differentially expressed in a variety of tumors compared with corresponding normal tissues, such as BRCA, CHOL, COAD, KICH, KIRC, KIRP, LICH, LUAD, and PRAD (Figure 3F). Taken together, these results suggested that the three hub lncRNAs associated with EMT are potential indicators for quantifying the malignant features of tumors.

### Prognostic value of Epithelial-mesenchymal transition-related LncRNA-based stratification

**FIGURE 4:**
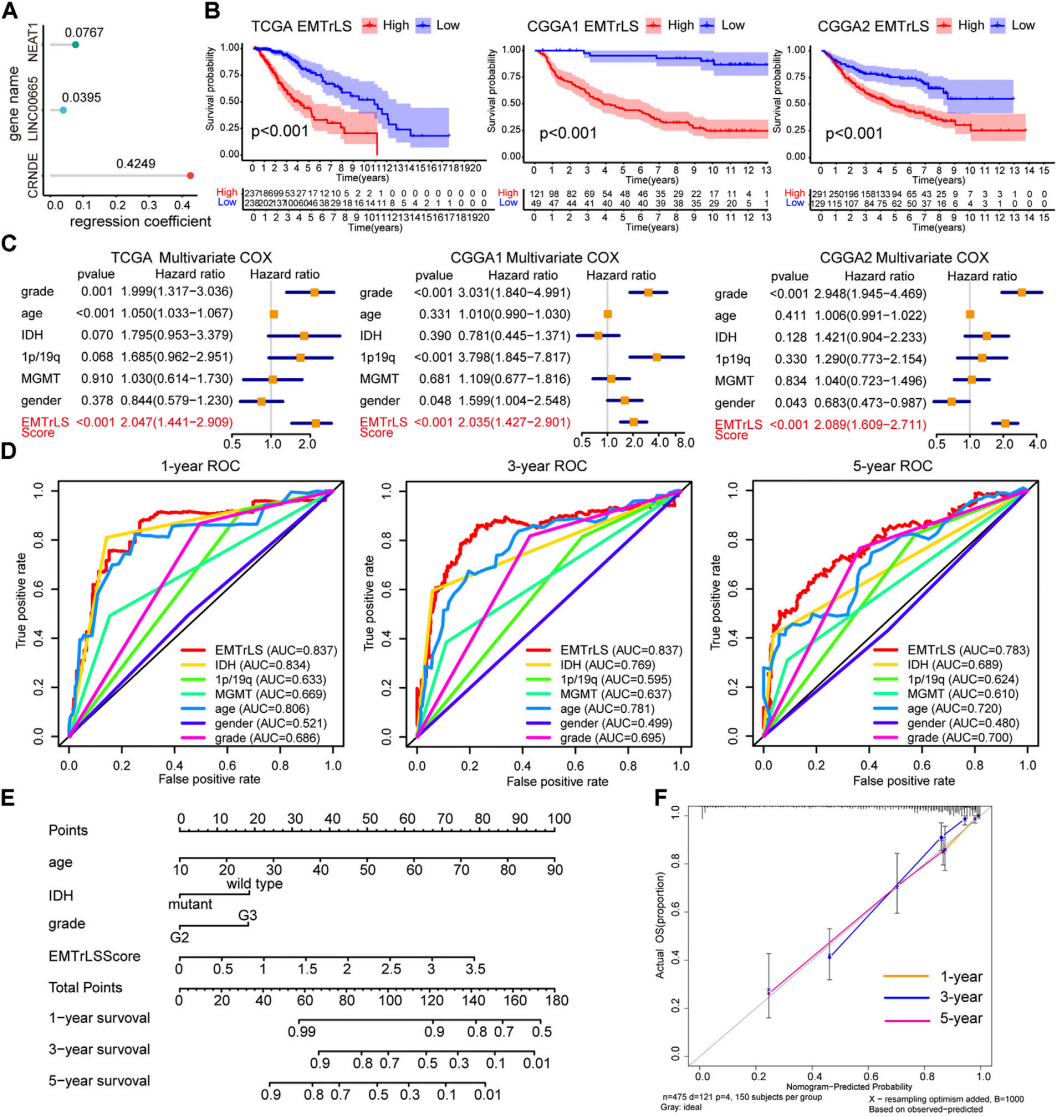
**(A)** LASSO regression coefficients of the three hub lncRNAs **(B)** K-M survival curves for EMTrLS-high and -low cohorts in the three datasets **(C)** Multivariate Cox regression analysis of EMTrLS scores, as well as WHO grade, age, IDH, 1p/19q, and MGMT status **(D)** ROC curves for the EMTrLS score and clinicopathological parameters including gender, grade, age, IDH, MGMT, and 1p/19q status in the TCGA dataset (one-, three-, and five years) **(E)** Nomogram constructed based on EMTrLS score, age, IDH, and grade for clinical determination **(F)** Calibration plots of a nomogram predicting one-, three-, and five-year survival probabilities.

Samples’ EMTrLS was quantified based on the expression of the three hub lncRNAs and their LASSO regression coefficients (Figure 4A). K-M survival curves showed that samples with increased EMTrLS scores had significantly decreased OS (*p* < 0.001) (Figure 4B). Univariate and multivariate Cox regression analyses suggested that EMTrLS could be an independent risk factor (Figure 4C, Supplementary Figure S2A). In addition, ROC curves were used to assess the sensitivity of prognostic indicators, showing that the one-, three-, and five-year AUC values for the EMTrLS score were greater than those of molecular markers such as IDH, 1p/19q, and MGMT, respectively. This result suggested a robust time-dependent predictive power of the EMTrLS (Figure 4D, Supplementary S2B,C). In view of the robust prognostic ability of EMTrLS, we constructed clinical nomogram plots to enable optimization of clinical treatment (Figure 4E). Meanwhile, the calibration plots validated the predictive power of the nomogram with powerful accuracy (Figure 4F). In conclusion, the EMTrLS constructed using the three hub lncRNAs was of prognostic significance.

### Correlation between EMTrLS and Epithelial-mesenchymal transition scores

**FIGURE 5:**
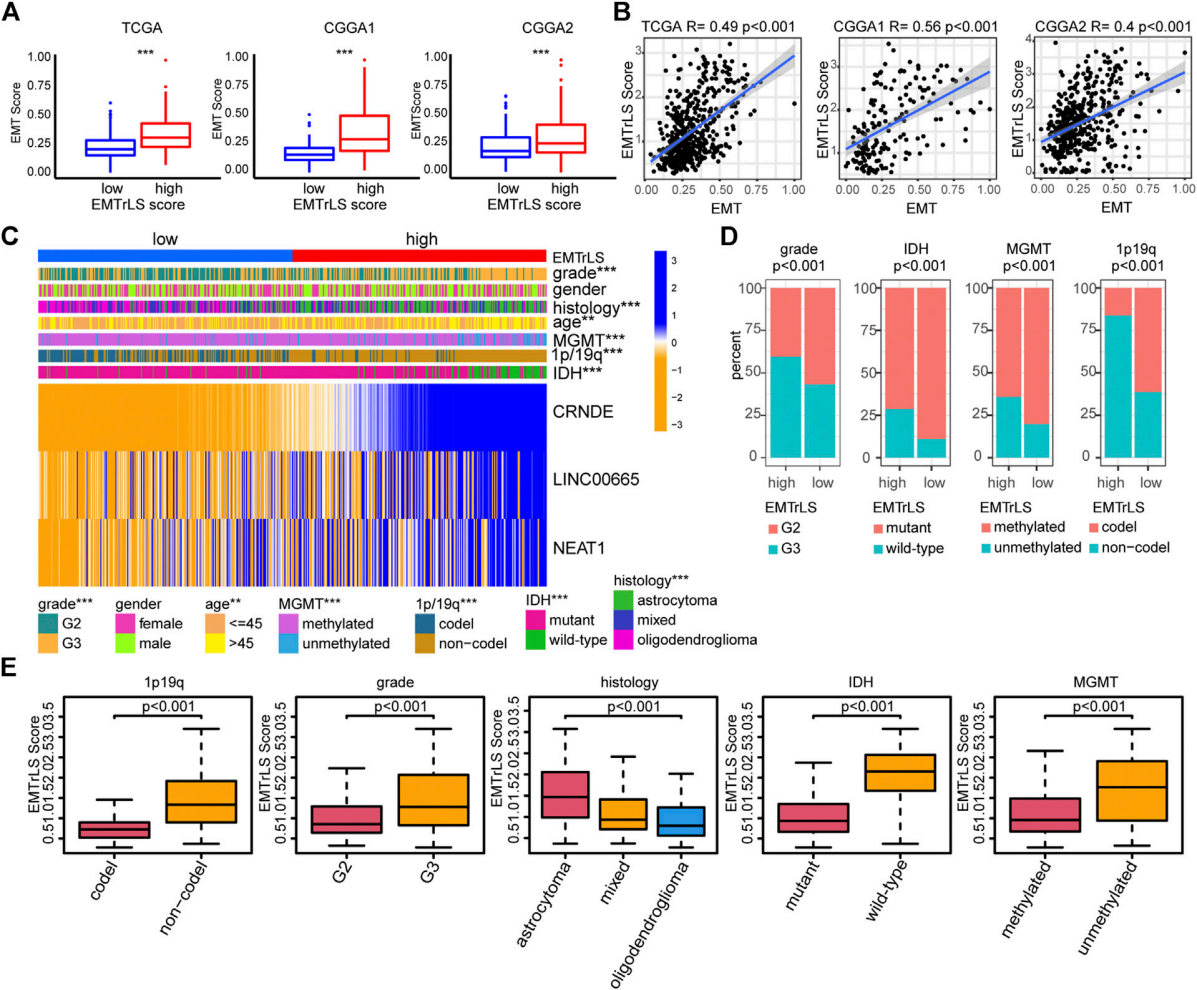
**(A)** The degree of EMT activation in the EMTrLS-high and -low cohorts in the three datasets **(B)** Spearman correlation analysis of EMT and EMTrLS scores **(C)** Heatmap showing the expression of hub genes and clinicopathological characteristics of two different EMTrLS groups based on the TCGA dataset **(D)** Stacked plots showing the distribution characteristics of clinicopathological parameters for the EMTrLS-high and -low cohorts including grade, IDH, MGMT, and 1p/19q status **(E)** Differences in EMTrLS scores for clinicopathological subgroups of the TCGA dataset, including 1p/19q, IDH, MGMT, grade and pathological histology (*p < 0.05, **p < 0.01, and ***p < 0.001)

To further validate the correlation between our constructed EMTrLS and tumor EMT status, we first explored the difference in EMT scores between the two LGG groups with high and low EMTrLS scores. The results showed that EMT scores were significantly increased in the EMTrLS-high samples, implying activation of EMT (*p* < 0.001) (Figure 5A). Furthermore, we performed a Spearman correlation analysis between EMTrLS and EMT scores, and the correlation coefficients were: 0.49, 0.56, and 0.4 in the TCGA, CGGA1, and CGGA2 datasets, respectively (*p* < 0.001) (Figure 5B). In summary, EMTrLS is associated with EMT activity.

### Association between EMTrLS and clinicopathological features

With the heatmap, we further demonstrated the expression of the three hub genes in EMTrLS and found that the expression of the three genes increased with the EMTrLS score (Figure 5C, Supplementary S3A,B). Furthermore, we found that patients with low EMTrLS scores were characterized by IDH mutation, WHO grade II, 1p/19q codeletions, and MGMT promoter methylation (*p* < 0.001) (Figure 5D). In addition, the EMTrLS scores also showed significant differences when IDH, WHO grade and 1p/19q status differed, which was perfectly validated by the two CGGA datasets. Notably, the EMTrLS scores also differed significantly by LGG pathology histology (*p* < 0.001) (Figure 5E, Supplementary S3C,D). In summary, EMTrLS can distinguish LGGs with different characteristics.

### Functional enrichment

**FIGURE 6:**
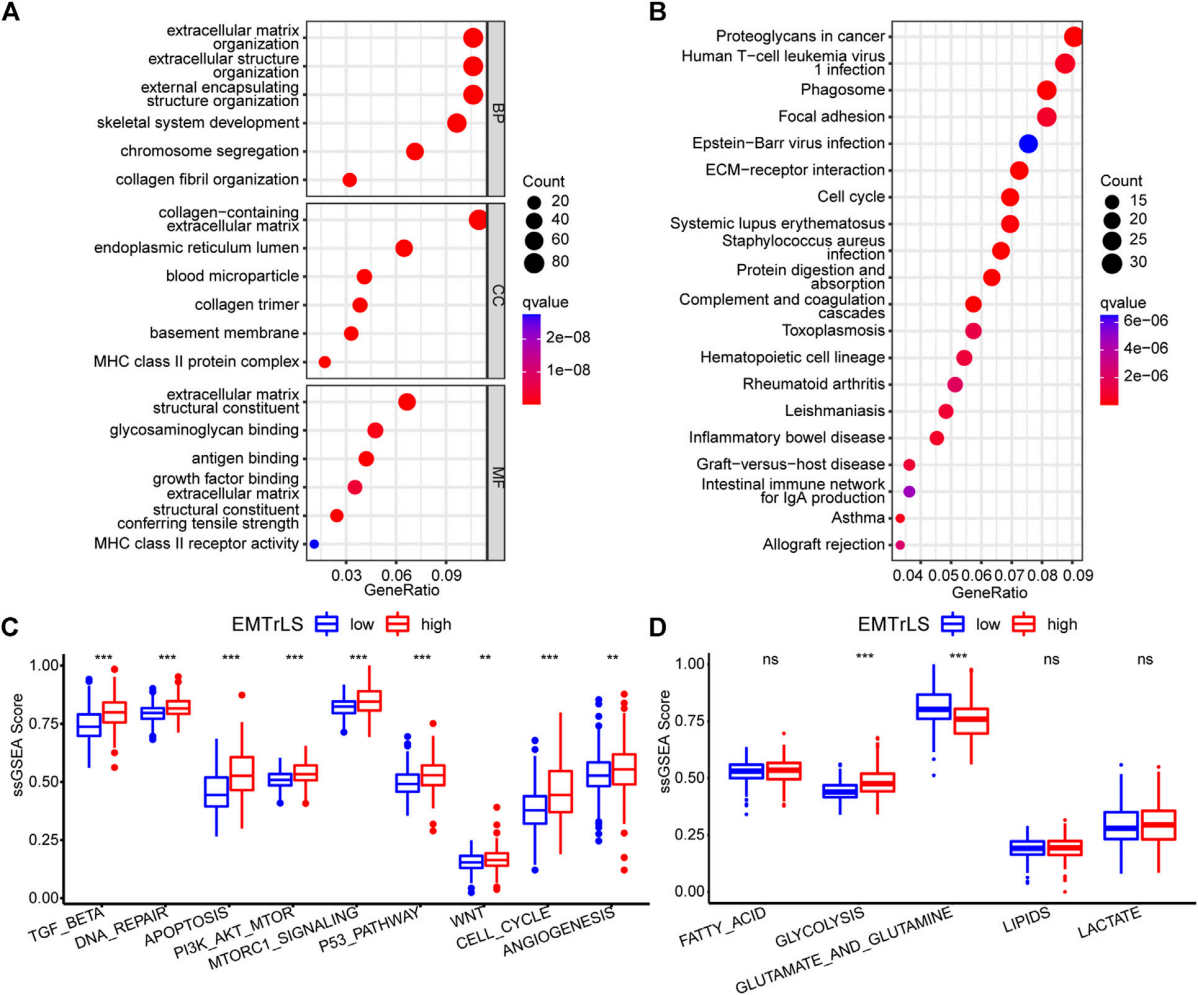
**(A,B)** Functional enrichment analysis of 912 upregulated genes in the EMTrLS-high subgroup was performed using GO analysis of BP, CC, MF, and KEGG **(C,D)** Analysis of 9 tumor-associated classical pathways and five metabolic pathways based on the ssGSEA algorithm for the TCGA dataset (ns means nonsignificant, *p < 0.05, **p < 0.01, and ***p < 0.001)

To clarify the functional differences associated with EMTrLS, we screened 1,252 differentially expressed genes between the EMTrLS-high and -low groups (Supplementary Table S6). GO and KEGG analyses were performed on 913 genes upregulated in the EMTrLS-high group. GO analysis revealed that pathways such as extracellular matrix, extracellular matrix structural constituent, and extracellular matrix organization were significantly upregulated. In addition, KEGG analysis highlighted that proteoglycans in cancer were upregulated in the EMTrLS-high group (Figures 6A,B). Interestingly, these genes upregulated in the EMTrLS-high group were also enriched in some immune-related pathways such as MHC class II protein completion and MHC class II receptor activity. This result suggested that there may be a potential association of EMTrLS with immune infiltration and immune function.

To further explore the tumor cell aggressiveness between the EMTrLS-high and -low groups, nine classical tumor pathways including the TGFβ pathway, DNA repair, apoptosis, PI3K-AKT-mTOR signaling, mTORC1 signaling, P53 pathway, WNT pathway, cell cycle, and angiogenesis were quantified by the ssGSEA algorithm. The scores of these pathways differed slightly across datasets but showed significant elevation in the EMTrLS-high group in at least two datasets. Collectively, EMTrLS characterized the malignant pathways associated with tumor development and metastasis (Figure 6C, Supplementary Figures S4A,B). In addition, we calculated the activation state of the metabolic pathways of LGG. The unlimited proliferative capacity and abnormal functional state of tumor cells often require a higher degree of glucose metabolism and protein metabolism to provide energy. The metabolic pathways of the glycolytic, glutamate and glutamine showed high activation in the EMTrLS-high group of all three datasets, indicating that EMTrLS is also related to the glycolytic pathways and the metabolic status of glutamate and glutamine in LGG (Figure 6D, Supplementary Figures S4C,D).

### Immune-related features

**FIGURE 7:**
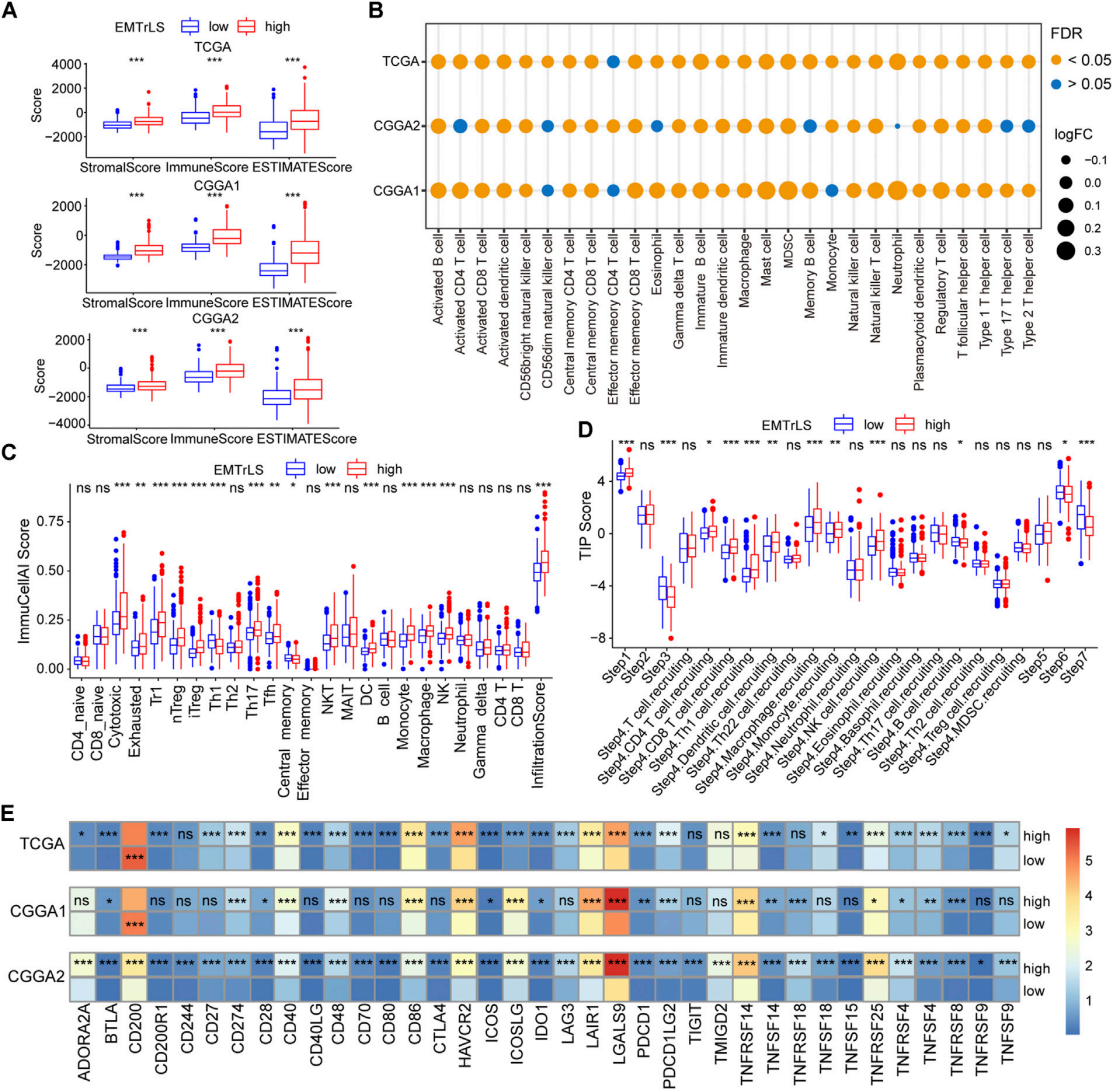
**(A)** The “estimate” algorithm calculates the degree of tumor immune infiltration, including immune, stromal, and estimate scores, based on the gene expression of the three datasets. Tumor purity of TCGA dataset **(B)** Calculation of immune infiltrated cells by ssGSEA algorithm based on the TISIDB summary of signature genes. The size of the bubble is positively proportional to the log (fold change) value (EMTrLS-high vs EMTrLS-low group), and the color represents the FDR q-value **(C)** ImmuCellAI online analysis of 24 immune infiltrating cells in TCGA tumor samples **(D)** Differences in antitumor immune processes between high and low EMTrLS score cohorts **(E)** Heatmap of immune checkpoint expression in different EMTrLS groups. The colors represent the mean values of individual gene expression (ns means nonsignificant, *p < 0.05, **p < 0.01, and ***p < 0.001)

Given the interplay between EMT and the tumor immune microenvironment, we further analyzed the association between EMTrLS and immune infiltration in LGG. The stromal, immune, and ESTIMATE score were higher in the EMTrLS-high group, suggesting a higher level of immune infiltration (Figure 7A). By calculating ssGSEA scores for 28 immune cells, we found increased levels of immune cell infiltration, including CD8 T cells, central memory CD4 T cells, gamma delta T cells, B cells, Treg cells, T follicular helper cells, type 1 T helper cells, dendritic cells, macrophages, mast cells, MDSCs, NK cells, and NK T cells (Figure 7B, Supplementary Figures S4E–G). Correspondingly, we similarly found elevated immune infiltration scores in the EMTrLS-high group using the ImmuCellAI calculation. In addition, this method yielded 24 immune cells, of which T-cell cytotoxicity, exhausted T-cells, NK cells, Th17 cells, Tregs, Th1 cells, dendritic cells, monocytes, and macrophages were also significantly increased in the EMTrLS-high group (Figure 7C, Supplementary Figures S5A,B). Macrophages can activate and induce EMT through the TGF-β, NF-κB pathway (Wei et al., 2019). This prompted us to further explore the correlation between EMT-related lncRNA and macrophage infiltration levels. The results showed that the Spearman correlation coefficients of EMTrLS scores with macrophages in the three datasets were 0.28, 0.33, and 0.26 (*p* < 0.001) (Supplementary Figure S5C).

It was interesting to note that both analysis methods showed a significant increase in the infiltration of immune cells with tumor-killing effects, in addition to immune cells such as macrophages, and Treg cells, which have the ability to suppress antitumor immunity, in the group with high EMTrLS scores. This result seems contradictory to the result that the EMTrLS-high group had a poorer prognosis. Therefore, we determined the antitumor immune differences between the different EMTrLS samples with the aid of the TIP algorithm. As a result, there was a significant increase in the third, sixth and seventh steps, which means that patients with low EMTrLS scores had a higher degree of T cell stimulation and activation, recognition, and killing of cancer cells (Figure 7D).

Furthermore, the differential expression of immune checkpoints and other molecules in the two LGG groups further explained the lower antitumor effect of the EMTrLS-high group. BTLA, CD274, CD28, CD40, CD48, CD86, HAVCR2, ICOS, ICOSLG, IDO1, LAIR1, LGALS9, PDCD1, TNFSF14, TNFRSF14, TNFRSF25, TNFRSF4, TNFSF4, and TNFRSF8 in the three databases were upregulated in the EMTrLS-high group. Activation of BTLA suppressed the function of CD8 cancer-specific T cells (Chen et al., 2019). The interaction of CD274 (PDL1) and PDCD1 (PD1) suppresses the cellular immune response of an organism, thus allowing tumor cells to evade surveillance and clearance by the immune system (Jiang et al., 2019). The increased expression of HAVCR2 (Bassez et al., 2021), LGALS9 (Wang et al., 2020), and LAIR1 (Peng DH et al., 2020) promoted the depletion and functional decline of T cells. In addition, ICOSLG (Iwata et al., 2020), and IDO1 (Zhai et al., 2018) promoted Treg cell activity and suppressed antitumor immunity. CTLA-4, which was upregulated in the high EMTrLS group, exerted a negative regulation of the immune response upon binding to the equally upregulated T cell costimulatory ligand CD86 (Chen and Mellman, 2017) (Figure 7E, Supplementary Figures S6A–C).

### EMTrLS-related genomic features

**FIGURE 8:**
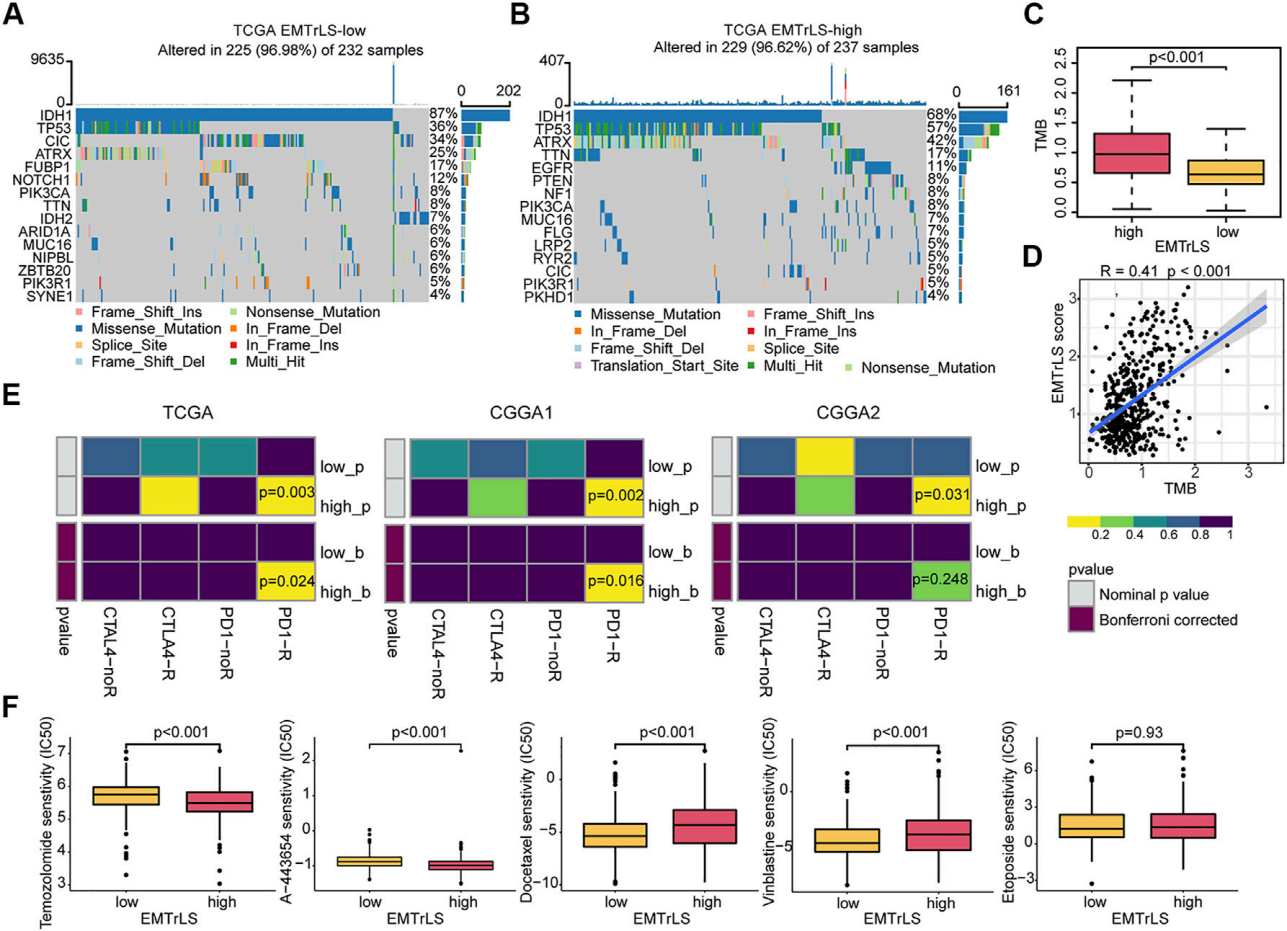
Gene mutations between **(A)** low and **(B)** high EMTrLS score samples (top 15) **(C)** Different EMTrLS samples have significantly different TMB **(D)** Correlation analysis of TMB and EMTrLS score **(E)** Prediction of the association of EMTrLS stratification with ICB responsiveness **(F)** Prediction of the IC50 values for different chemotherapeutic agents in high and low EMTrLS cohorts of TCGA, including temozolomide, A-443654 (AKT inhibitor), docetaxel, etoposide, and vinblastine.

Mutations in important genes have a role in patient prognosis and treatment outcome. We compared gene mutations between the two samples groups. As a result, the overall number of mutation frequencies was very similar; however, the specific mutated genes were very different. The top 5 mutations with highest frequencies in samples with low EMTrLS scores were IDH1 (88%), TP53 (36%), CIC (34%), ARTX (25%), and FUBP1 (17%), while the top 5 mutations with highest frequencies in samples with high EMTrLS scores were IDH1 (68%), TP53 (57%), ATRX (42%), TTN (17%), and EGFR (11%) (Figures 8A,B). Increased mutations in TP53, an important regulator of cell growth, proliferation, and damage repair, often predict poor prognosis (Donehower et al., 2019). ATRX is a histone chaperone protein that loads histones onto telomeres and maintains heterochromatin (Qin et al., 2022). Loss of ATRX activates the immunosuppressive transcriptome and immune escape mechanisms in LGG cells.

Evidence from multiple sources suggests that higher TMB is associated with better outcomes after ICB treatment, particularly with PD-1 inhibitors (Chalmers et al., 2017). Stratified analysis showed that EMTrLS significantly differentiated the TMB values of LGG samples (Figure 8C). In addition, we found a positive correlation between TMB and EMTrLS score (cor = 0.41, *p* < 0.001) (Figure 8D). This result implied that there is a potential correlation between EMTrLS scores and ICB immunotherapy outcomes that warrants further exploration.

### ICB responsiveness and chemotherapy outcomes based on EMTrLS score stratification

In clinical applications, ICB is a promising therapeutic approach, resulting in long-lasting survival benefits for patients with melanoma and hepatocellular carcinoma (Postow et al., 2015). With the submap algorithm, we predicted the effect of ICB treatment in patients with a different EMTrLS. As a result, LGGs with high EMTrLS were more likely to respond to anti-PD1 therapy (TCGA *p* = 0.003, Bonferroni-corrected *p* = 0.024; CGGA1 *p* = 0.003, Bonferroni-corrected *p* = 0.016; CGGA2 *p* = 0.031, Bonferroni-corrected *p* = 0.248), suggesting that EMTrLS-based stratification has the potential to predict the response to anti-PD1 therapy (Figure 8E).

There is a significant difference in response to TMZ, which is a first-line chemotherapeutic agent administered after surgery in LGG patients (Omuro and DeAngelis, 2013). Accordingly, the “PRRophetic” package was employed to predict the response to chemotherapeutic agents among different EMTrLS groups. The results showed that the IC50 estimates were lower in the high-EMTrLS population (*p* < 0.001) (Figure 8F, Supplementary Figures S6D,E). In addition, we included other therapeutic agents including A-443654 (Akt inhibitor), docetaxel, etoposide, and vinblastine. The results showed that the IC50 estimates of A-443654 were higher in the EMTrLS-low group in all three datasets (*p* < 0.001). The IC50 estimates of docetaxel were lower in the EMTrLS-low group of the TCGA and CGGA1 datasets (*p* < 0.001). This showed that the EMTrLS score can guide the clinical treatment of different LGG groups. And A-443654 and doxorubicin also have potential application inthe treatment of glioma.

## Discussion

To our knowledge, this is the first application of machine learning to mine multiple datasets to identify the most well characterized EMT-related lncRNAs. In this study, we finally obtained three hub lncRNAs and used them to construct a scoring system to explore the characteristic patterns of EMT-related lncRNAs in LGG tumor cells. The stratified analysis revealed that samples with different EMTrLS possessed different clinicopathological parameters and EMT activation statuses. Another feature of the EMTrLS-high group was the activation of tumor malignancy-related pathways, as well as the activation of glycolytic pathways, and glutamate and glutamine metabolic pathways. In addition, EMTrLS distinguished the heterogeneity of immune infiltration and antitumor immunity in LGG samples. Notably, the EMTrLS-high subgroup was not only more sensitive to TMZ treatment but also more responsive to PD1 blockade therapy. Moreover, coupled with the powerful prognostic predictive power of the EMTrLS scoring system, it has the potential to guide more effective and precise treatment.

Our study revealed that abnormal states of EMT are present in LGG tumor tissues and correlated with malignant clinicopathological features, which may modulate the aggressiveness and treatment resistance of glioma cells (Pastushenko and Blanpain, 2019). Although, the study of genes associated with EMT is currently popular among researchers inthe field of oncology. The functions of single lncRNAs such as TCL6 (Kulkarni et al., 2021), AP000695.4 (Liang et al., 2018), and RP11-390F4.3 (Peng PH et al., 2020), in the EMT process, have been discussed and confirmed in detail. Using bioinformatics approaches, many interesting EMT-related genes have been mined and predictive models have been constructed, including colorectal, bladder and clear cell renal cell carcinomas (Cao et al., 2020; Zhong et al., 2020; Yang et al., 2021). The construction of these risk models reveals that EMT-related genes can be used as molecular markers for optimal tumor treatment and have powerful prognostic capabilities. However, there still exists a large number of lncRNAs that have not yet been tapped identified, resulting in incomplete understanding of EMT-related lncRNAs. Therefore more comprehensive work is needed to confirm the expression and function of EMT-related lncRNAs in LGG. This would be helpful for a deeper understanding of the molecular features and aggressiveness of low-grade gliomas.

In our study, a series of algorithms such as univariate Cox regression, RF, and LASSO regression, were used to identify the hub lncRNAs CRNDE, LINC00665, and NEAT1. The expression of CRNDE regulates cancer cell proliferation, migration, invasion, and apoptosis through multiple signaling pathways, such as WNT/β-catenin (Yu et al., 2017), PI3K/AKT (Liu et al., 2017), and mTOR (Wang et al., 2015) signaling pathways. LINC0066 is aberrantly expressed in a variety of human cancers and acts as an oncogene or tumor suppressor gene (Zhu et al., 2022). Silencing LINC00665, which is associated with poor prognosis in gliomas, suppressed the expression of markers of EMT (Lu et al., 2021). The last hub lncRNA NEAT1 promotes glioblastoma development and progression through the EGFR/NEAT1/EZH2/WNT/β-catenin axis (Chen et al., 2018). In addition, NEAT1 also promotes gliomagenesis through the mTOR signaling pathway (Yu et al., 2021). From these studies, it is clear that CRNDE, LINC00665, and NEAT1 are highly oncogenic and inextricably linked to EMT. This also explained that the signature models constructed from these three hub genes were able to describe the activation status of multiple tumor malignancy-related pathways. Importantly, no study has emerged that incorporates these three genes simultaneously in statistical model construction. Our constructed EMTrLS score demonstrated a stable correlation with EMT in multiple datasets and described the malignancy and aggressiveness of LGG with a strong predictive prognostic value. Of course, it cannot be said that these three pivotal lncRNAs can fully characterize all EMT-related lncRNAs. We hope that our study can help characterize EMT-related lncRNAs and provide a new way of thinking about tumorigenesis and development.

The characterization based on the three hub lncRNAs constructs was able to not only quantify the extent of EMT and malignant tumor pathway activation but also reveal its potential correlation with abnormal tumor metabolism. It is possible that dysregulation of WNT signaling control induces EMT, while increasing glucose consumption and lactate production through activation of pyruvate carboxylase (PC) gene expression (Lee et al., 2015). In addition to this, the hypoxic microenvironment of gliomas may be another cause of the relationship between glucose metabolism and EMT abnormalities. Hypoxia-inducible factors (HIFs) increase the expression numerous glycolytic enzymes and can also activate EMT through various pathways, including TGF-β, Notch, PI3K/AKT, WNT/β-catenin, and NF-κB (Tirpe et al., 2019).

The process of EMT stimulates the production of cytokines and chemokines in the TME to promote the infiltration of immune cells. In turn, TME is an effective inducer of EMT in tumor cells. The mutual maintenance of these two phenomena alters the expression and activity of various cell types that accumulate in the mesenchyme, particularly in response to various immune cell subtypes that influence tumor progression (Suarez-Carmona et al., 2017; Dongre and Weinberg, 2019). However, few studies have elucidated the potential mechanisms of EMT-related lncRNAs and immune infiltration in LGG. We applied multiple immunoassays to explore immune cell infiltration in LGG and determined the association with EMTrLS stratification. Interestingly, we observed a significant macrophage and Treg infiltration in the EMTrLS-high group, accompanied by an increased infiltration of immune cells with tumor-killing effects, such as CD4 T cells and CD8 T cells. It may seem paradoxical that there was a higher degree of infiltration of immune cells with antitumor function in EMTrLS-high samples with poor prognosis. However, some studies have shown that CD8 T cells and CD4 T cells, also lead to loss of E-cadherin expression in epithelial cells, accompanied by increased expression of vimentin and ZEB1 (EMT markers) (Santisteban et al., 2009; Goebel et al., 2015). Although the mechanism is unclear, cytokines such as IL-6 may facilitate this process (Chen et al., 2017). Furthermore, given the association of EMTrLS with EMT activation, it is not surprising that EMTrLS scores can be quantified to some extent for antitumor immune cells. Moreover, these immune cells that attack cancer cells are often overwhelmed by various immunosuppressive cells that also have increased infiltration in the EMTrLS-high group, such as MDSCs, macrophages, and Tregs. They can directly inhibit the antitumor function of T cells and NK cells and aid tumor progression (DeNardo and Ruffell, 2019; Togashi et al., 2019; Veglia et al., 2021). Notably, another stratification study of EMTrLS revealed that the EMTrLS-high group with poorer prognosis had overexpression of ICP, such as PD-1, PD-L1, CD86, HAVCR2, and ICOS. This further explains the reason why the antitumor immune function of the EMTrLS-high group was not well performed. The significant infiltration of immunosuppressive cells and overexpression of ICP may be the key mechanism of immune escape in EMTrLS-high samples.

TMB has been shown to be a reliable biomarker for tumor selection for ICB treatment. The exact mechanism is not known, but most patients who benefit from treatment tend to have a high TMB (Rizvi et al., 2015; Chan et al., 2019). In this study, we found a potential correlation between EMTrLS scores and TMB. EMTrLS stratified analysis indicated that the EMTrLS-high group had a higher TMB and may be a potential gaining group for immunotherapy. This is similar to the results of our other study. A subclass mapping algorithm we used revealed that the EMTrLS-high group was more sensitive to PD1 blockade treatment. These results illustrate the potential of EMTrLS to guide more effective anti-PD1 therapy. However, due to the lack of LGG cohorts treated with ICB, the predictive power of EMTrLS requiresfurther validation in more prospective trials. In predicting chemotherapy response, we found that the EMTrLS-high cohorts were more sensitive to TMZ and AKT inhibitor drugs. However, the mechanisms underlying the correlation between EMTrLS and chemotherapy response need to be further investigated. Likewise, a large number of drug response experiments are needed to confirm these predictions.

In conclusion, we constructed a novel EMTrLS with the help of machine algorithms, which can comprehensively assess the malignancy and prognosis of individual patients and provide new insights for diagnosis and clinical treatment decisions. However, it is undeniable that our study has some limitations. We should further confirm the specific mechanisms by which the three hub lncRNAs affect EMT through *in vivo* and *in vitro* experiments, and further validate their regulatory effects on tumor immune function and immune cells. In addition, our developed EMTrLS scoring system for the assessment of antitumor treatment sensitivity needs to be further verified in prospective studies and chemotherapy drug sensitivity assays. The EMTrLS model should include more clinical factors in practical clinical applications to improve predictive accuracy. We will incorporate these efforts into future studies.

## Data Availability

The original contributions presented in the study are included in the article/Supplementary Material, further inquiries can be directed to the corresponding authors.
