# Supplementary figures and images for: Machine learning-based screening of an epithelial-mesenchymal transition-related long non-coding RNA signature reveals lower-grade glioma prognosis and the tumor microenvironment and predicts antitumor therapy response
Source: Front Mol Biosci. 2022 Aug 26;9:942966. doi: 10.3389/fmolb.2022.942966 (PMC9459009; doi:10.3389/fmolb.2022.942966)

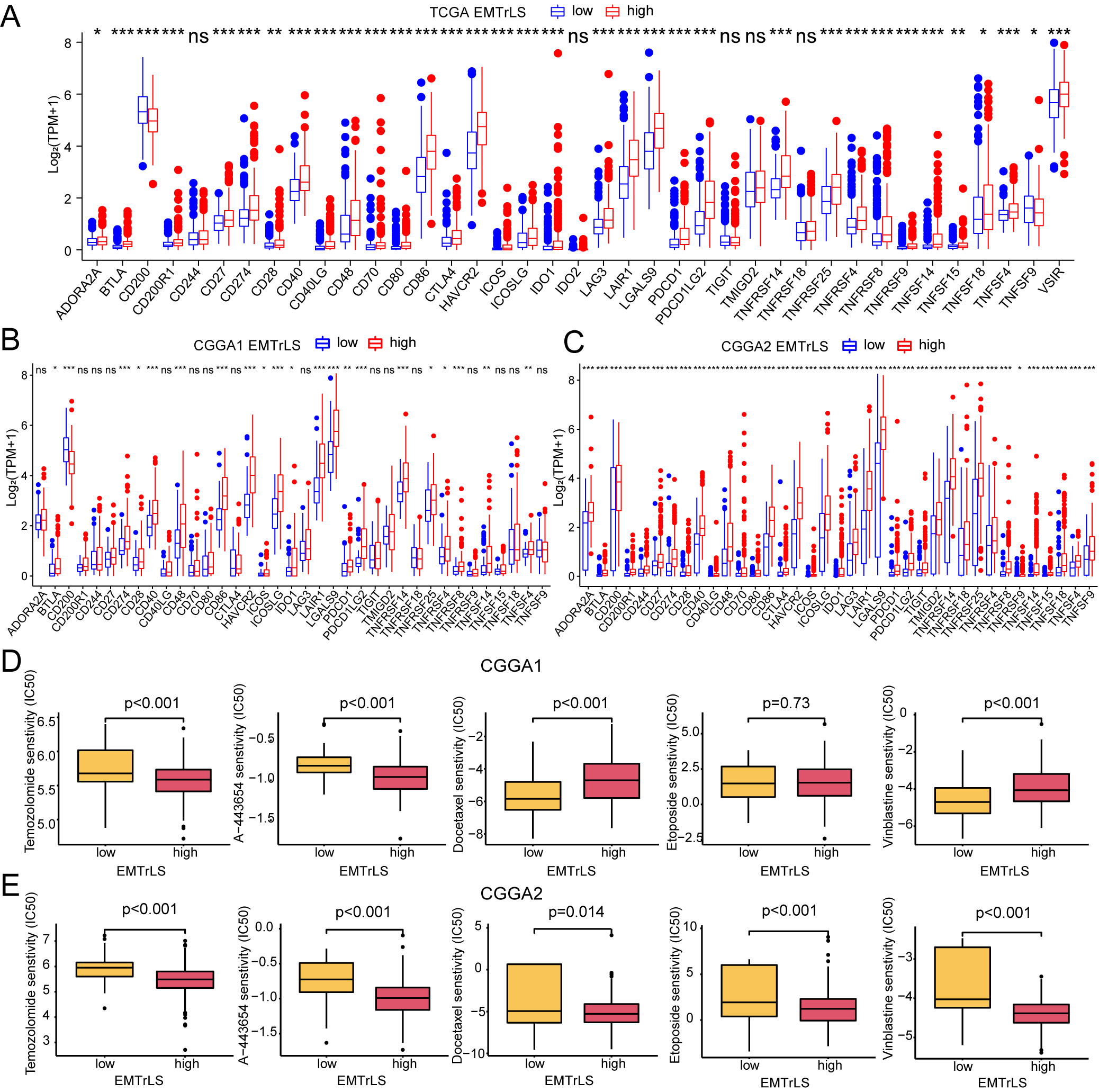

Supplement: Supplementary file 1 [file Image6.TIF]

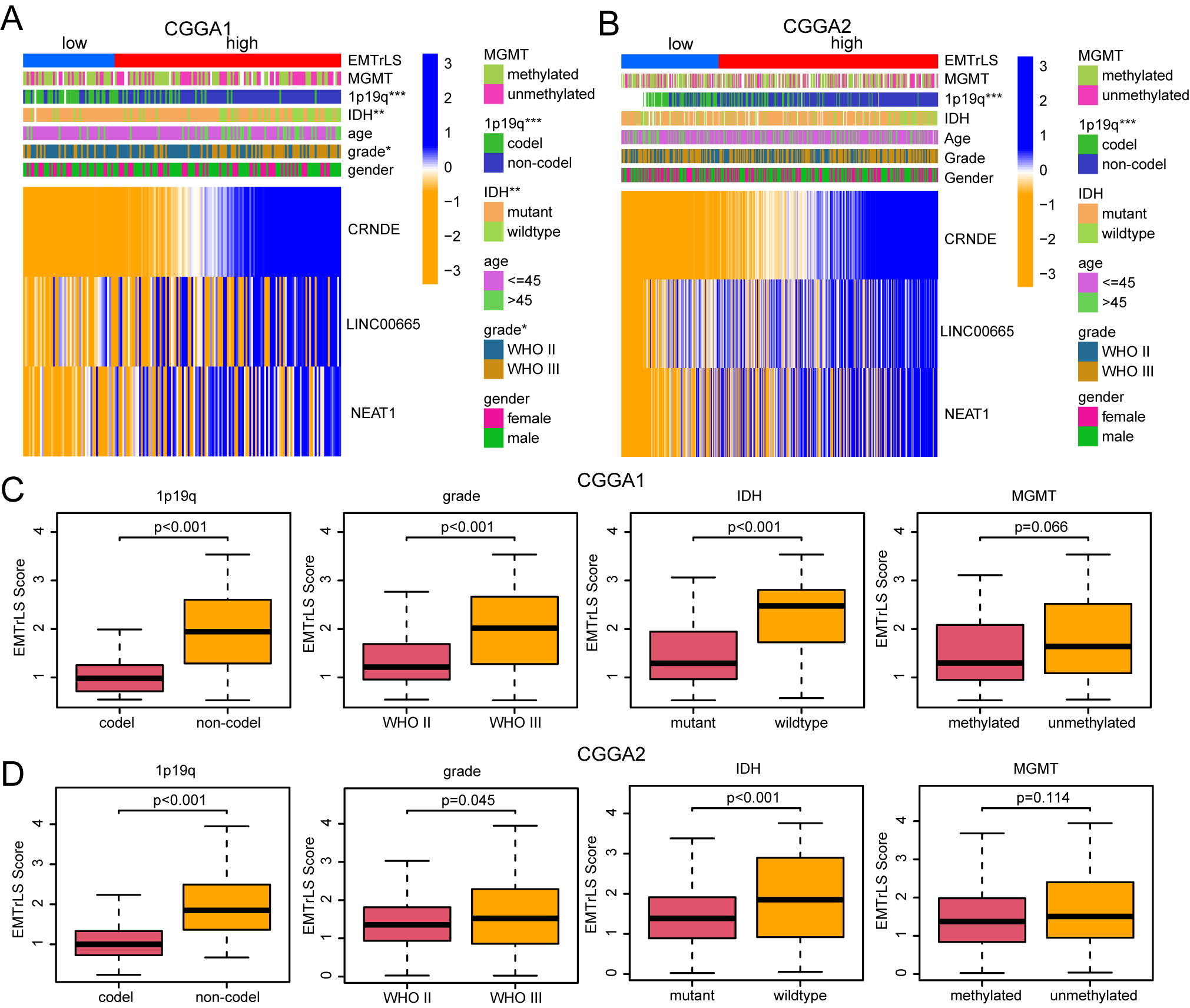

Supplement: Supplementary file 2 [file Image3.TIF]

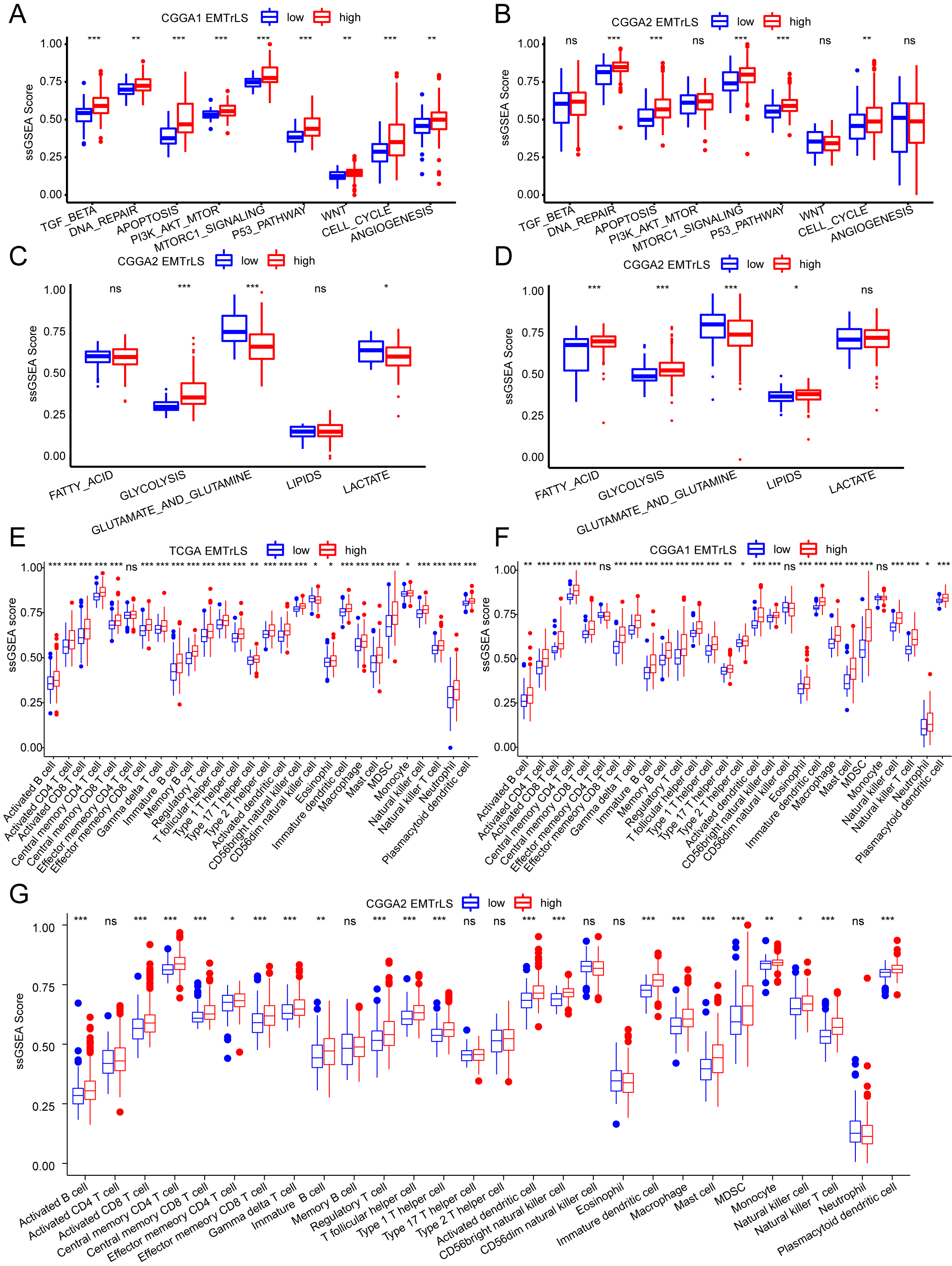

Supplement: Supplementary file 3 [file Image4.TIF]

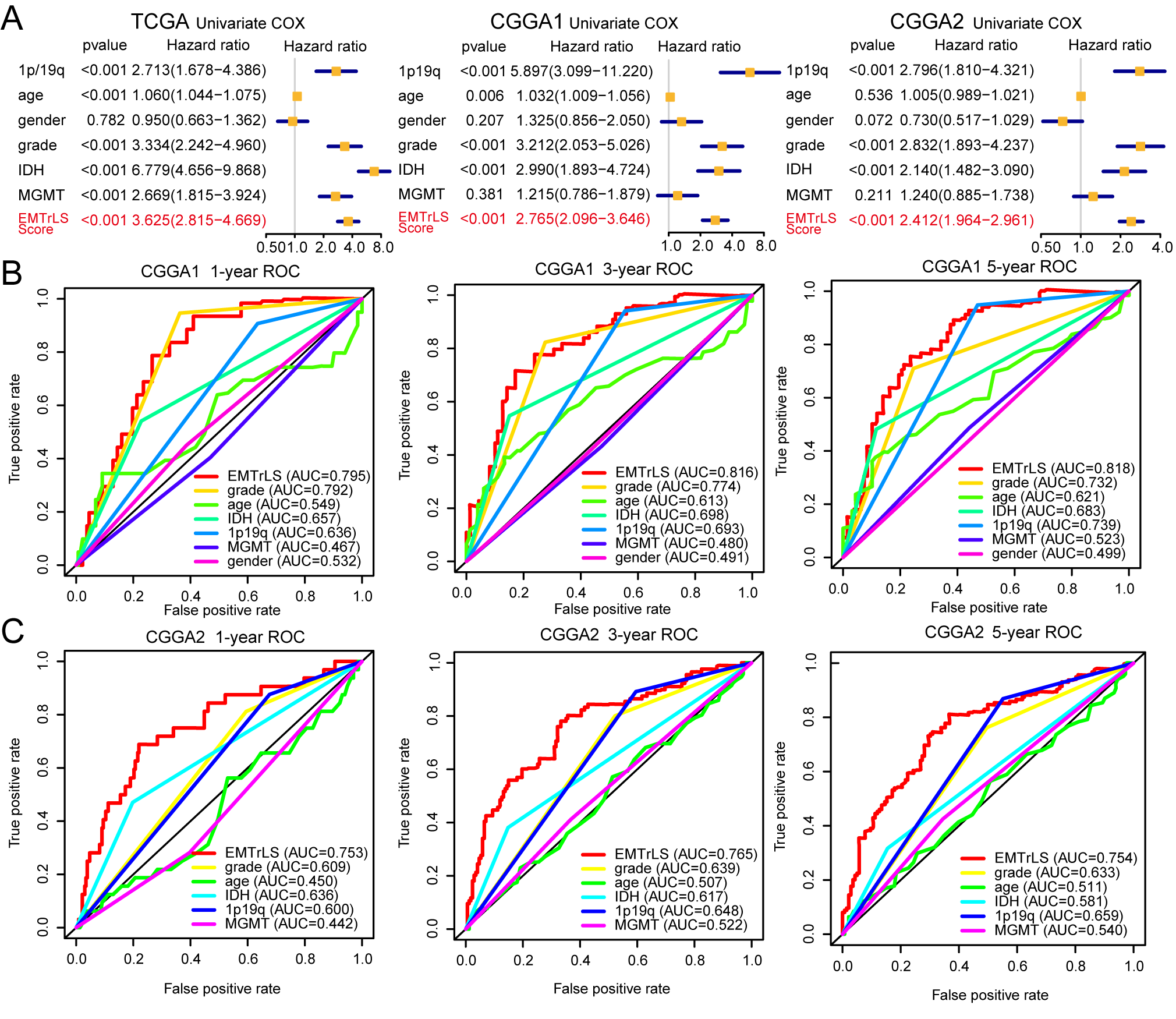

Supplement: Supplementary file 4 [file Image2.TIF]

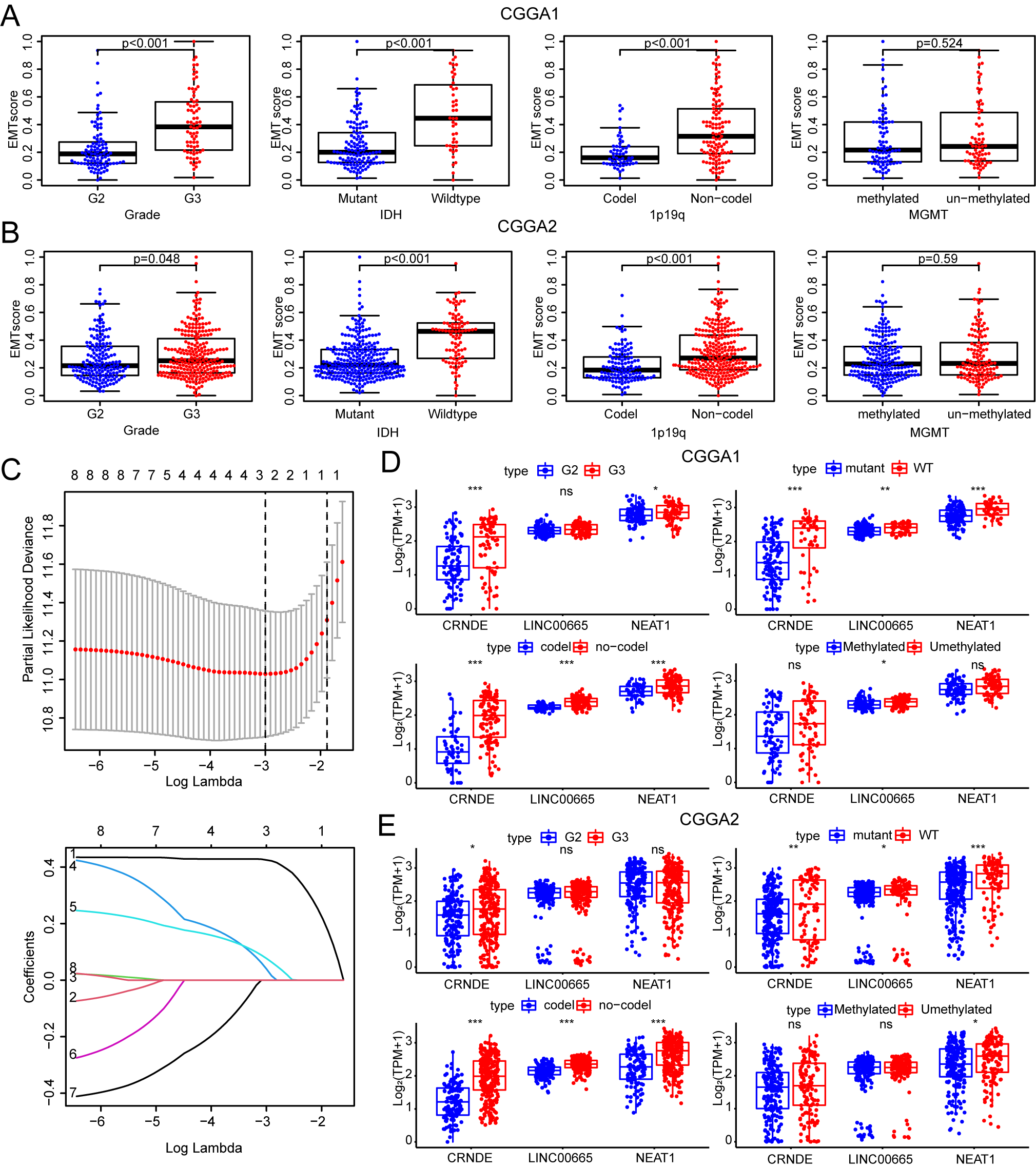

Supplement: Supplementary file 5 [file Image1.TIF]

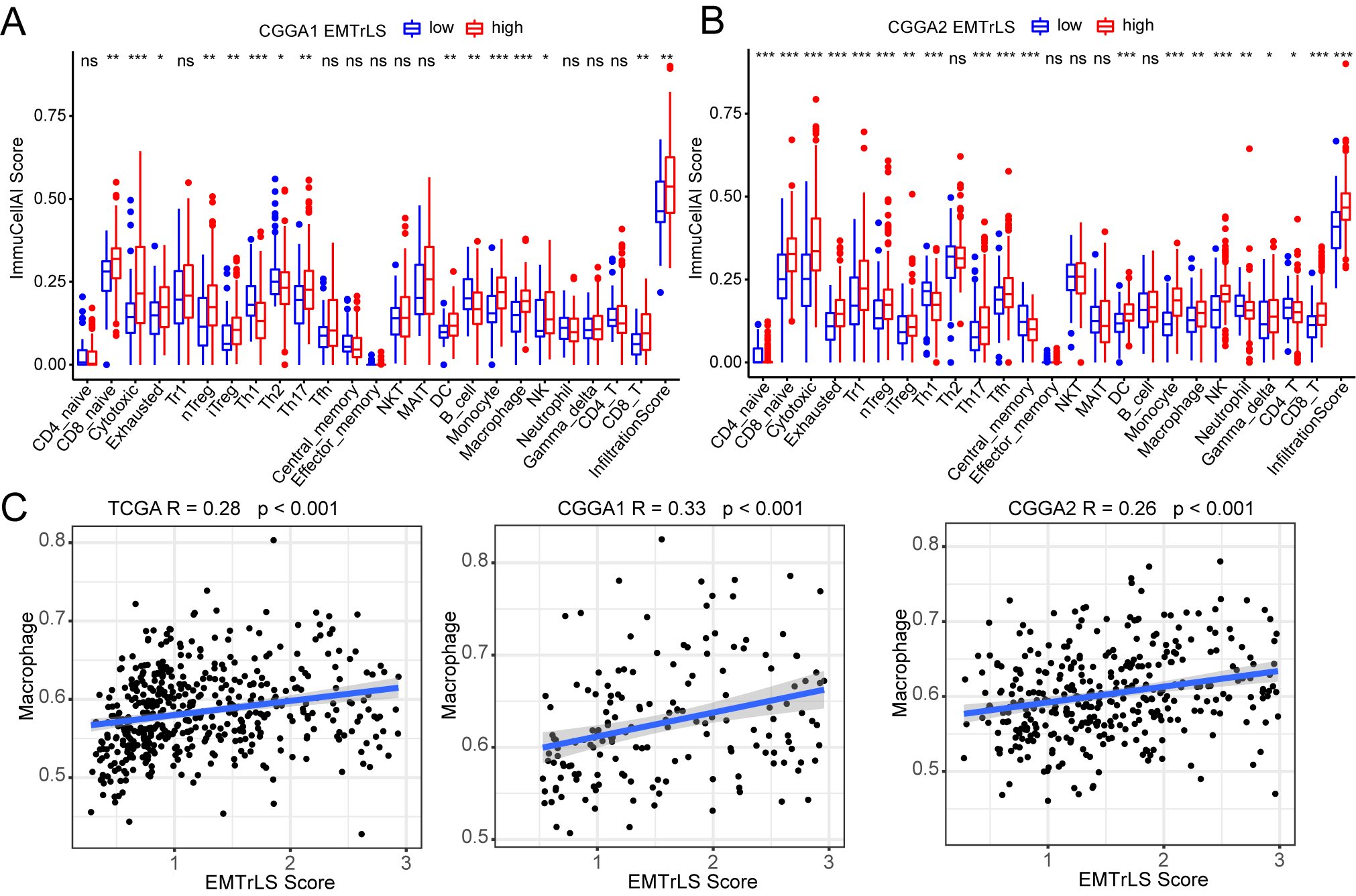

Supplement: Supplementary file 7 [file Image5.TIF]
